# Patient outcomes following discharge from secure psychiatric hospitals: systematic review and meta-analysis

**DOI:** 10.1192/bjp.bp.114.149997

**Published:** 2016-01

**Authors:** Seena Fazel, Zuzanna Fimińska, Christopher Cocks, Jeremy Coid

**Affiliations:** **Seena Fazel**, MD, **Zuzanna Fimińska**, MSc, University of Oxford, Oxford, UK; **Christopher Cocks**, MD, Department of Psychiatry, University of New South Wales, Sydney, Australia; **Jeremy Coid**, MD, Wolfson Institute of Preventive Medicine, Queen Mary University London, London, UK

## Abstract

**Background**

Secure hospitals are a high-cost, low-volume service consuming around a fifth of the overall mental health budget in England and Wales.

**Aims**

A systematic review and meta-analysis of adverse outcomes after discharge along with a comparison with rates in other clinical and forensic groups in order to inform public health and policy.

**Method**

We searched for primary studies that followed patients discharged from a secure hospital, and reported mortality, readmissions or reconvictions. We determined crude rates for all adverse outcomes.

**Results**

In total, 35 studies from 10 countries were included, involving 12 056 patients out of which 53% were violent offenders. The crude death rate for all-cause mortality was 1538 per 100 000 person-years (95% CI 1175–1901). For suicide, the crude death rate was 325 per 100 000 person-years (95% CI 235–415). The readmission rate was 7208 per 100 000 person-years (95% CI 5916–8500). Crude reoffending rates were 4484 per 100 000 person-years (95% CI 3679–5287), with lower rates in more recent studies.

**Conclusions**

There is some evidence that patients discharged from forensic psychiatric services have lower offending outcomes than many comparative groups. Services could consider improving interventions aimed at reducing premature mortality, particularly suicide, in discharged patients.

Over the past two decades, there have been large increases in the numbers of secure psychiatric hospital beds, which some have argued amounts to a reinstitutionalisation of psychiatric patients.^1^ Costs per patient are substantially more in such hospitals, with some estimates of £152 000 per year per patient in the UK at low secure institutions and £273 000 in high secure hospitals^2^ and an estimated overall budget of over £1 billion.^3^ In England, this is equivalent to 19% of the overall mental health budget and represents its largest single component. However, the evidence for patient benefit in such hospitals is limited. Single studies have followed cohorts of discharged patients in a number of countries, and there is a need to synthesise these reports, present information on all adverse outcomes and provide some comparative information for public health and policy to contextualise these findings. Therefore, we have conducted a systematic review of studies that have tracked patients after secure hospital discharge for criminal behaviour, readmission to psychiatric hospital and mortality.

## Method

We searched for studies that described long-term outcomes of forensic psychiatric patients in 11 computer-based literature indexes: PubMed, Google Scholar, PsycINFO, JSTOR, Global Health, Medline, Web of Knowledge, DART-Europe, E-thesis portal, Networked Digital Library of Thesis and Dissertations, and ProQuest Dissertations and Theses (the latter four for theses and dissertations). To supplement this, we scanned the reference lists from each of the articles, and followed-up on citations of the papers identified. Non-English articles were translated. No language or country restrictions were imposed. The search was performed from the start of the database until 13 March 2013. For the database search, we used combinations of keywords relating to patients (patient, forensic, mental disord*, mental illness, psychiatric disord*, psych*, felon*), institutions (low, high, medium, secur*, special, hosp*) and outcome (outcome, mort*, rehosp*, death, readm*, reconvict*, reoffend*, recidi*, rearrest, repeated offend*). For reporting of the meta-analysis, PRISMA guidelines were followed (as many of the studies were evaluating services).^4^ For assessing the quality of studies included, we used the Newcastle–Ottawa Quality Assessment Scale for Cohort Studies.^5^

For inclusion in the systematic review, studies had to meet the following criteria: (a) primary studies; (b) investigations that followed up patients discharged from any secure hospital, including low-, medium- or high-security institutions; and (c) reported on outcomes for death, suicide, repeat offending (including violent behaviour, contact with police, rearrests or reconvictions) or readmission to hospital (including returning to the same institution or admission to another psychiatric hospital).

Studies were excluded if they met the following criteria: (a) a validation study for a risk assessment tool; (b) evaluation of an intervention; and (c) did not provide data that would allow for calculation of rates. We excluded studies of risk assessment and interventions, as those are conducted on a select group of patients who give consent to participate in a study, and could yield a biased sample. Our review focused exclusively on observational studies. In the case of duplicate publications, we selected the publication with the most information. Where needed, authors were contacted for clarification. Data extraction was performed independently by Z.F. and C.C., any discrepancies were resolved by discussion, and when consensus could not be reached, the differences were resolved by the project supervisor S.F. We extracted data on background characteristics of the samples in order to study factors associated with heterogeneity: year of publication, geographical location, sample size, percentage male, age, percentage with convictions, index offence, percentage violent, legal category according to the English and Welsh Mental Health Act 1983 (which involves two main categories: mental illness and a legal category of psychopathic disorder (that is practice equates to severe personality disorder). The updated version of the Act in 2007 removed the legal classification of psychopathic disorder), admission duration, time in the community and mean follow-up period. If studies reported different causes of death, we extracted all data.

We calculated crude rates (CRs) for all-cause mortality, suicides, readmissions and reoffending by using number of events (*N*_e_) and person-years at risk (*PY*_total_), following the methods outlined in a recent meta-analysis of released prisoners:^6^

$$\mathrm{CR}=N_{e}∕{\mathrm{PY}}_{\text{total}}$$

We calculated person-years at risk based on the number of patients (*N*_p_) and median period of patient follow-up (*PY*_med_):

$${\mathrm{PY}}_{\text{total}}=Np{\mathrm{PY}}_{\mathrm{med}}$$

We used the Wilson's method to calculate 95% confidence intervals around those estimates because the asymptotic method produces intervals that can extend below zero.^7^

We performed random-effects meta-analyses on crude rates to calculate pooled estimates for all-cause mortality, suicides, readmissions and reoffending. Random-effects models incorporate an estimate of between-study heterogeneity into the calculation of the common effect and give relatively similar weights to all studies.^8^ We assessed heterogeneity by using *I*^2^, which describes the percentage of variation across studies that is because of heterogeneity rather than chance, and does not inherently depend on the number of studies considered.^9^ Values 25%, 50% and 75% are taken to indicate low, moderate and high levels of heterogeneity respectively.^9^ Potential sources of heterogeneity were investigated by arranging groups of studies according to potentially relevant characteristics, and by meta-regression analysis. Factors examined were geographical location (England and Wales *v.* other countries, as 9684, or 80% of the participants were based in England and Wales), age, admission duration, proportion with mental illness, proportion with personality disorder, proportion with prior convictions, proportion whose index offence was violent (homicide/attempted homicide, non-fatal violence, sexual offence), year of publication and national crime rate. Categorical variables explored were region (England and Wales *v.* other countries), age (⩽35 *v.* >35 years), mental illness (⩽80% *v.* >80%), psychopathic disorder (⩽10% *v.* >10%), previous convictions (⩽60% *v.* >60%), violent offence (⩽30% *v.* >30%) and sexual offences (⩽9% *v.* >9%). Cut-off scores were chosen to ensure that the groups were approximately even. If there were less than ten studies, meta-regression was not conducted as statistical power was limited.^10^ All analyses were performed in Stata statistical software package, version 12 using the commands metan (for random effects meta-analysis), and metareg (for meta-regression).

We conducted an additional analysis that compared the estimated reoffending rates in released prisoners (nominator) with reoffending rates in forensic patients (denominator) to calculate rate ratios and 95% confidence intervals. We used where possible released prisoners from a similar age band to the forensic patients. Information about reoffending rates of prisoners was obtained from the Ministry of Justice or equivalent of each country.^11–13^

### Comparisons

A number of clinical and forensic populations were considered as possible comparisons in this study, for example community psychiatric patients, prisoners and mentally disordered offenders sentenced to community-based interventions (i.e. not in-patient treatment). Four computer-based databases were searched to identify eligible studies: PubMed, Google Scholar, Global Health, and Web of Knowledge. We used key words relating to study participants (disor*, offend*, pris*, felon*, patient*, community), mental illness (schizo*, psych*, mental ill*, psychiatric ill*) and outcomes (mortality, suicide, readm*, rehosp*, reoff*, rearrest*, reconv*).

## Results

We identified 35 relevant studies published between 1982 and 2013 (online Fig. DS1 and online Table DS1).^14–44^ The total sample included 12 056 patients (75% male), with a mean age of 34.5 years, and of which 53% were violent offenders, and 18% had a previous conviction (the latter being based on 10 reports). The average length of admission was 3 years. Overall, 18 investigations were from England and Wales (*n* = 9684), 4 from the USA (*n* = 428), 3 from Sweden (*n* = 297), 2 from Australia (*n* = 222), 2 from New Zealand (*n* = 240), 2 from Italy (*n* = 209), 2 from Canada (*n* = 362), and 1 each from Japan (*n* = 489) and Norway (*n* = 125). All but nine studies reported average age, but not uniformly with average age at admission, discharge, start of follow-up or at index offence being reported. Average follow-up ranged from 1.5 to 13.6 years for mortality, 1.8 to 9.4 years for readmissions and 1.5 to 13.6 years for reoffending. Studies used a variety of sources to collect follow-up information on patients, including hospital records, coroners' records, and regional and national databases.

### All-cause mortality and suicide

#### All-cause mortality

There were eight publications reporting on mortality in nine cohorts (*n* = 2226).^15–21,40^ Two additional studies reported solely suicides (*n* = 4502).^14,44^ The total number of deaths was 368, of which 143 (39%) were suicides.

**Fig. 1:**
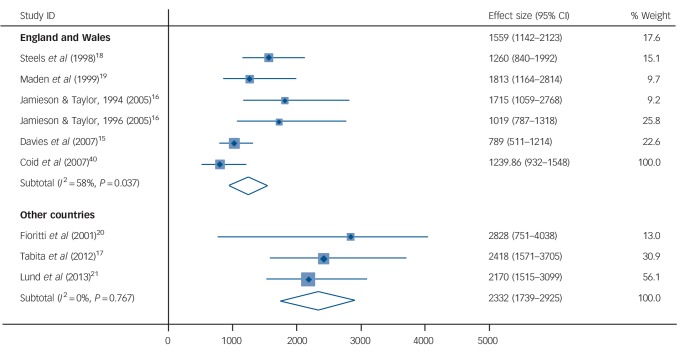
Crude mortality rates of discharged forensic psychiatry patients for all-cause mortality in England and Wales compared with other countries. Rates are per 100 000 person-years. Weights are from random-effects analysis.

All-cause crude death rates (CDRs) ranged from 789 to 2828 per 100 000 person-years (online Table DS2). The pooled estimate for all-cause CDR was 1538 (95% CI 1175–1901) per 100 000 person-years (*I*^2^ = 71%, 95% CI 41–85%). Subgroup analysis revealed some influence of location on all-cause death rates, with studies based in England and Wales reporting lower mortality (CDR = 1240, 95% CI 932–1548) compared with those from other countries (CDR = 2332, 95% CI 1739–2925) (Fig. 1). Meta-regression was not conducted because of a limited number of samples. As a result of insufficient information in the included studies, standardised mortality ratios (SMRs) were not reported.

#### Suicide

Six studies reported suicide outcomes.^14,15,17,19,40,44^ The CDR was 325 (95% CI 235–415) per 100 000 person-years (*I*^2^ = 19%, 95% CI 0–64%) (Table DS2). Meta-regression was not conducted because of the limited number of samples.

#### Comparisons

Six studies reporting on mortality in comparative groups were identified: released prisoners, mentally disordered offenders and community psychiatric patients (*n* = 7 667 645) (Table 1).^6,21,45–50^ Rates ranged from 850 to 3344 per 100 000 person-years for all-cause mortality, and from 155 to 561 per 100 000 person-years for suicide. The highest all-cause mortality rate was reported in mentally disordered offenders sentenced to non-custodial sanctions,^21^ and for suicide, it was all people with criminal justice history.^50^

**Table 1:** Mortality rates for populations comparative with forensic psychiatric patients

Study	Country	Patient population	Crude all-cause mortality rate per 100 000 (95% CI)	Suicide rate per 100 000 (95% CI)
Pratt *et al* (2006)^47^	UK	Released prisoners	–	155 (140–171)

Kariminia *et al*^a^ (2007)^49^	Australia	Recently released prisoners admitted to the prison psychiatric hospital	–	300 (61–538)

Brown *et al* (2010)^45^	UK	Schizophrenia	1772 (1523–2063)	–

Dutta *et al* (2012)^46^	UK	Psychosis	1417 (1292–1554)	–

Kinner *et al* (2011)^48^	Australia	Prisoners	874 (818–934)	156 (141–172)

Webb *et al* (2011)^50^	Denmark	All people with criminal justice history	–	561 (549–574)

Zlodre & Fazel (2012)^6^	Seven countries	Recently released prisoners (meta-analysis)	850 (815–884)	169 (123–214)

Webb *et al* (2012)^51^	Denmark	Violent and sexual criminal offenders	–	163 (136–191)

Lund *et al* (2013)^21^	Sweden	Mentally disordered offenders sentenced to prison	1274 (746–2168)	300 (138–648)

Lund *et al* (2013)^21^	Sweden	Mentally disordered offenders sentenced to non-custodial sanctions	3344 (1923–5754)	–

Current review	England/Wales	Forensic patients	1240 (932–1548)	–

Current review	Non-England/Wales	Forensic patients	2332 (1739–2925)	–

Current review	Overall estimate	Forensic patients	1538 (1175–1901)	325 (235–415)

a. Kariminia study has a 6 month follow-up.

### Readmissions

**Fig. 2:**
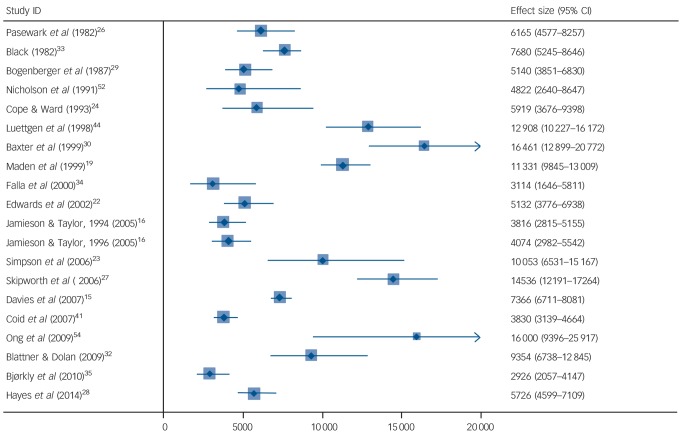
Psychiatric hospital readmission rates for discharged forensic psychiatric patients per 100 000 person-years. Weights are from random-effects analysis.

In total, 20 studies reported on hospital readmissions in 21 cohorts (*n* = 3522).^15,16,19,22–24,26–30,32–35,40,44,52–54^ The total number of patients readmitted was 1171. Crude readmissions rates (CRARs) ranged from 2926 to 16 461 readmissions per 100 000 person-years (online Table DS3 and Fig. 2). The pooled estimate for CRAR was 7208 (95% CI 5916–8500) per 100 000 person years, with substantial heterogeneity (*I*^2^ = 92%, 95% CI 89–94%). In individual variable meta-regression analyses, studies with a greater proportion of patients classified under the Mental Health Act as having a mental illness reported a higher readmission rate (β = 108.6, s.e.(β) = 54.9, *P* = 0.070), whereas the reverse was found for psychopathic disorder (β = −181.4, s.e.(β) = 90.6, *P* = 0.070). In addition, patients who had a longer admission were more likely to be readmitted, although this association did not reach statistical significance (β = 90.9, s.e.(β) = 46.4, *P* = 0.076). In models combining combinations of these factors, none of them retained statistical significance. None of the other characteristics (including age, type of index offence or previous convictions) significantly explained heterogeneity.

#### Comparisons

Five studies reporting on readmissions of community patients, mentally disordered veterans and offenders with mental disorders (not guilty by reason of insanity) treated in an out-patient programme were identified (*n* = 36 317) (online Table DS4).^55–59^ Rates ranged from 3838 to 55 555 per 100 000 person-years, with the highest readmission rate reported for offenders with mental disorders treated in an out-patient programme.^59^

### Reoffending

**Fig. 3:**
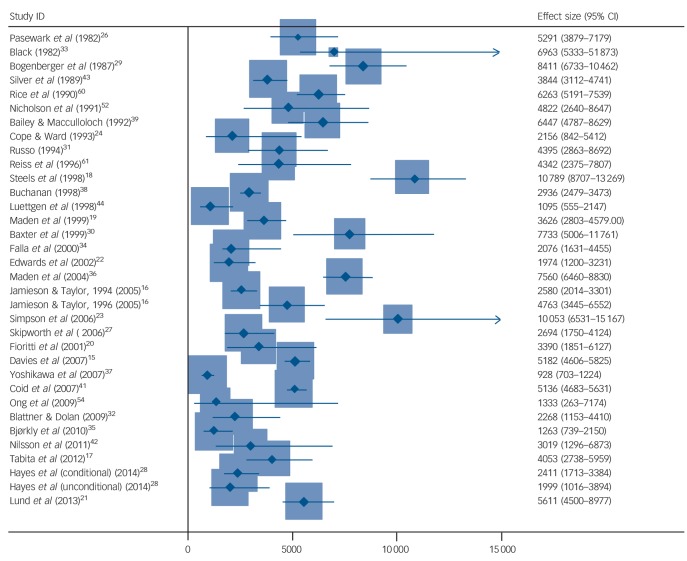
Repeat offending rates for discharged forensic patients per 100 000 person-years. Weights are from random-effects analysis. The two outliers (combined no. reoffenders = 6) are not shown.

In total, 30 papers reported on criminal outcomes.^15–18,20,21,23,24,26–34,36–40,42–45,52–53,60,61^ Crude reoffending rates ranged from 0 to 24 244 per 100 000 person-years (online Table DS5). The pooled estimate was 4484 per 100 000 person-years (95% CI 3679–5287), with very high heterogeneity (*I*^2^ = 95%, 95% CI 94–96%) (Fig. 3), partially explained by the higher rates in studies conducted earlier (β = −101.1, s.e.(β) = 43.3, *P* = 0.026). Neither age, geographical region, type of index offence, duration of admission, Mental Health Act category or history of in-patient psychiatric treatment reached statistical significance.

#### Comparisons

Ten studies reporting on repeat offending in different samples were identified: released prisoners, offenders with personality disorders, mentally disordered offenders and offenders with mental illness (*n* = 696 757) (Table 2).^12,13,21,25,62–64^ Rates ranged from 4535 to 36 964 per 100 000 person-years, with the highest reoffending rate reported for prisoners released on probation.

**Table 2:** Reoffending rates for populations comparative with forensic patients

Study	Country	Population	Crude reoffending rate (95%CI)
Home Office (2003)^64^	England and Wales	Released prisoners with original sentence of 1–4 years	27 003 (26 303–27 703)

Home Office (2003)^64^	England and Wales	Released prisoners with original sentence of 5–10 years	17 987 (15 964–19 768)

Langan & Levin (2002)^13^	USA	Released prisoners	15 627 (15 548–15 706)

Swedish National Council for Crime Prevention (2011)^11^	Sweden	Released prisoners (21–39 years)	15 176 (14 944–15 408)

Fazel & Yu (2011)^63^	Eight countries	Offenders with psychosis (meta-analysis)	4535 (4269–4801)

Yu *et al* (2012)^25^	Seven countries	Offenders with personality disorder (meta-analysis)	7954 (7651–8258)

Lund *et al* (2013)^21^	Sweden	Mentally disordered offenders sentenced to non-custodial sanctions	7246 (4992–10 405)

Lund *et al* (2013)^21^	Sweden	Mentally disordered offenders sentenced to prison	5426 (4202–6981)

Ministry of Justice (2013)^12^	England and Wales	Offenders (mean age 30–34) (violent and non-violent offenders)	27 217 (26 891–27 544)

Ministry of Justice (2013)^12^	England and Wales	Prisoners released on probation	36 964 (36 401–37 530)

Current review	Eight countries	Forensic patients	4484 (3679–5287)

#### Violent reoffending

**Fig. 4:**
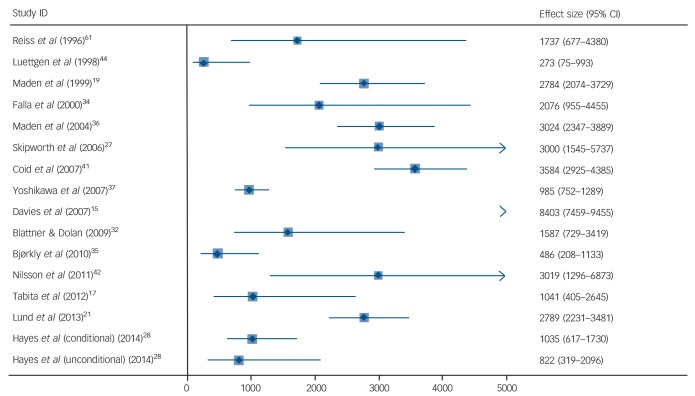
Repeat violent offending rates for discharged forensic patients per 100 000 person-years. Weights are from random-effects analysis.

Fifteen studies reported violent reoffending in forensic patients as outcome (online Table DS6).^15,17,19,21,27,28,32,34–37,41,42,44,61^ Crude reoffending rates ranged from 273 per 100 000 person-years to 8403 per 100 000 person-years. Pooled estimate was 3902 (95% CI 2671–5187) with substantial heterogeneity (*I*^2^ = 97%, 95% CI 96–98%) (Fig. 4). Neither age, diagnosis, geographical region, type of index offence, duration of admission, history of in-patient psychiatric treatment, nor year of publication reached statistical significance.

#### Reoffending rates compared with prisoners

**Fig. 5:**
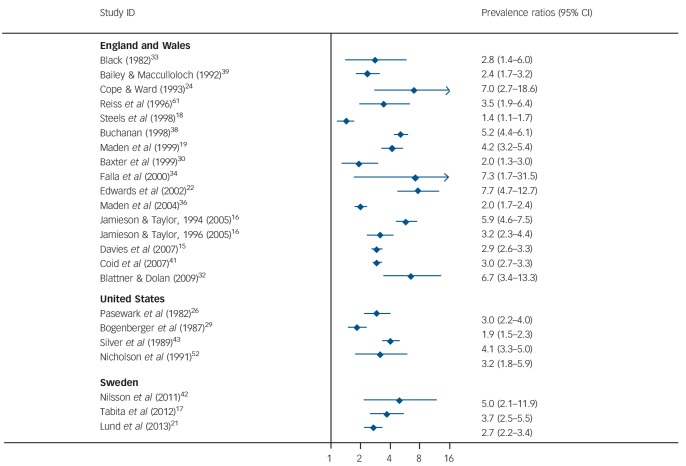
Prevalence ratios comparing reoffending rates of released prisoners with forensic psychiatric patients. Weights are from random-effects analysis. Prevalence ratios above 1 mean that rates of reoffending are higher in prisoners than in forensic psychiatric patients matched by age-band where possible.

We calculated the ratio of reoffending rate in prisoners with the reoffending rate in discharged forensic psychiatric patients using the same country and similar year for a comparative prisoner population of the same gender and, where possible, using similar age bands (ages 30–34) (Fig. 5). Prevalence ratios were one or above indicating that rates of prisoner reoffending were higher than in forensic psychiatric patients. The prevalence ratios ranged from 1.4 to 7.7 in UK studies, 1.9 to 4.1 in the USA and 2.7 to 5.0 in Sweden.

## Discussion

This review of 35 studies investigated rates of three adverse outcomes for psychiatric patients discharged from secure hospitals, namely mortality, readmission to hospital and repeat offending. The last of these is arguably the most important measure of benefit because risk of reoffending and violent behaviour had determined hospital admission in secure settings. We therefore calculated how these rates compare with repeat offending rates among prisoners from the same countries as made up the individual studies. Finally, we have provided a range of other comparisons for all these outcomes in order to contextualise the results.

### Mortality rates

Our main findings are that first, mortality rates among discharged forensic patients are high in both absolute and relative terms, with rates between 789 and 2828 per 100 000 patient-years. This compares, for example, with a rate of 850 in a recent review of all released prisoner studies,^6^ but is similar to studies of patients with predominantly schizophrenia-spectrum disorder (CDR = 1417).^46^ The latter suggests that it is the mental illness component of being in secure care, rather than anything specific to the forensic setting, that contributes to the increased mortality risk.

The possible reasons for this increased mortality risk are likely to be the same as those described in general psychiatric populations. These include increased prevalence of unhealthy lifestyle behaviours,^65^ such as physical inactivity, poor diet and importantly high rates of smoking and substance use.^66^ Side-effects of some psychotropic medications are relevant as they are associated with weight gain and type 2 diabetes.^67^ Another series of factors that contributes to the high levels of physical illness is the poor access to such services for psychiatric patients that may be related to poor insight from patients about the need for care but also stigma by certain health professionals. To address the increased mortality risk, a number of behavioural interventions are currently being trialed,^68^ and have been shown to reduce weight, although their effects on mortality are currently unknown. Further, anti-smoking treatments should be introduced including smoking-free hospitals and nicotine replacement therapies. The judicious use of psychotropic medication will be part of this, avoiding high doses where possible and polypharmacy.^69^

Interestingly, subgroup analysis found lower mortality rates in studies conducted in the English and Welsh samples compared with the rest of the world (which were made up mainly of studies from the USA, Sweden and other high-income countries). A possible explanation is there are better developed aspects of service provision (for example more community forensic psychiatry) in England and Wales, such as liaison with primary care. The possibility that services are more effective in England and Wales needs further research, as identifying the components in forensic services that reduce mortality risk will have wider implications. Nevertheless, absolute rates of suicide and mortality were high and secure hospitals should review all preventable deaths among their patients. Furthermore, death by suicide, where available, did not include open verdicts and are likely to be underestimates.

### Readmission rates

A second finding was that rates of readmission to hospital varied markedly from 2926 to 16 641 per 100 000 patient-years. We were not able to identify many comparative observational studies that reported on readmission rates for psychiatric patients, and therefore any conclusions about how these rates compare with general psychiatric services in the same countries are difficult. One potentially relevant explanation for the high rates of variation between the studies related to the relative proportions of mental illness and personality disorder (the latter identified using the legal category of ‘psychopathic disorder’, which usually equates to severe personality disorder) in the samples – the higher the rate of mental illness, the higher the rate of readmission. Another explanation is that in certain countries, mental health legislation facilitates compulsory recall to hospital following discharge and is at the discretion of the supervising physician. Readmission to hospital, however, is a specific outcome, and research reporting on other markers of function and quality of life in discharged forensic patients, including supported employment rates and symptom scores, is necessary. In addition, the studies reported in this review do not indicate whether readmission was to a forensic or a general hospital, and future research should separate these outcomes.

### Reoffending rates

Finally, we investigated repeat offending rates. This was the outcome where the most information was available, and we found rates varied between 0 and 24 244 per 100 000 patient-years. We were unable to find explanations for the heterogeneity between studies. This was expected as the determinants of reoffending are likely to be complex, many of which are unmeasured and interact. However, we did show that compared with reoffending rates for general prisoners matched by age, forensic patients had lower rates of repeat offending. In addition, we provided a range of other comparison groups, and the rates reported in this study were lower than these. Many of these comparisons are problematic as patients admitted to secure hospitals have committed more serious offences, and stay in hospital longer than equivalent groups. Therefore, we compared reoffending in individuals with violent index offences, and with prisoners with longer sentences as comparators, and we also investigated rates of violent reoffending and compared such rates with prisoners (online Table DS7). The latter is arguably more clinically important. Even with these comparisons, rates of repeat offending were lower in forensic patients.

Explanations for these differences in repeat offending may lie in the following. First, patients admitted to a secure hospital are highly selected and clinical staff in some jurisdictions have discretion on the basis of probable response to treatment. In contrast, prisons must accept all offenders sentenced by the courts. Thus, when considering the lower reoffending rates among discharged patients, it is important to consider the contrasting characteristics of prisoner populations in terms of their criminal careers, psychopathology, and both intensity and length of surveillance and social support following release into the community. Admission to a secure hospital in most countries follows serious violent and sexual offending, including homicide. This represents a very small proportion of all offenders processed by the criminal justice system. Serious offenders have lower rates of reoffending than those convicted of acquisitive, drug and minor violent offences, the latter characterising the majority of sentenced prisoners. Criminal careers research also shows that the latter subgroup have the highest rates of recidivism and specialism.^70^ Furthermore, the number of violent offences correspond to the number of acquisitive offences observed over the criminal career.^70^ This means that discharged patients with similar characteristics, including early-onset persistent offending, may be at similar risk of recidivism. However, for those with late-onset offending, with few previous convictions, those whose violence is exclusively against family members and where offending is temporarily associated with acute symptoms of severe mental illness, the probability of reoffending is low. These factors are characteristic of many patients in secure hospitals. The probability is further reduced by ongoing treatment and provision of supportive aftercare, particularly when mandated by mental health legislation.

### Implications

In conclusion, two main implications follow from our findings. First, there is some evidence that patients discharged from forensic psychiatric services have lower rates of repeat offending than many comparative groups. Second, such services could consider improving interventions that would reduce premature mortality in their discharged patients. These could take the form of follow-up care and better organised and coordinated services that comprehensively address the complex causes of mortality (including accidental deaths) instead of focusing on a single cause.^50^

### Limitations

Our study has a number of limitations. First, source of admission (court *v.* community) was not reported in most studies. The composition of a sample might have accounted for some of the heterogeneity between studies, but this could not be explored. Second, patients came from a range of institutions, which will have different admission criteria, and offer various treatments. Additionally, since the studies included in this review are from 1982 to 2013, admission criteria and available treatments will likely have changed over time, thus making comparisons between cohorts difficult. Third, most included studies did not report patient location at discharge, so ascertaining what proportion of the patients in each study reached the community is not straightforward. If patients are transferred to other hospitals, then reoffending rates are likely to be underestimated. Fourth, patients unconditionally discharged are difficult to trace, therefore some were lost to follow-up, meaning that some of the adverse events might have gone unreported and are underestimated. Fifth, we excluded studies of solely risk assessment instruments. Based on a recent review of the use of these tools,^71^ these studies are very heterogeneous in design, and, in research contexts, such tools constitute an intervention and may alter outcomes. Sixth, although effort has been made to ensure that patients are not double counted (for example studies with duplicate samples were excluded, and data were extracted from the studies that provided most information), there was an overlap between some studies coming from the UK.^15,36,41^ The overall number of patients who were double counted was approximately 180 (or 1.5% of the total review sample). Finally, there are some limitations associated with the quality of the studies included. The majority were retrospective, and used information on risk factors from case notes and outcomes from various official databases. Although a retrospective study has certain advantages over a prospective one including duration of follow-up, the quality and breath of patient information depends entirely on the quality and accuracy of clinical records kept. Moreover, official sources, such as the Offenders Index, used by all of the studies based in England and Wales, have their own limitations (Table DS1). First, the Offenders Index has a 2-year lag between charges and convictions; second, it is estimated that 9% of criminal records are missing from the Offenders Index,^39^ so the offending estimates are underestimates.

In summary, we have provided a systematic review and meta-analysis of outcomes of patients discharged from forensic psychiatric services. As such services have increased their patient numbers and costs in recent years, these findings should assist in their development in the UK and other countries.
